# African American English intensifier *dennamug*: Using twitter to investigate syntactic change in low-frequency forms

**DOI:** 10.3389/frai.2022.683104

**Published:** 2023-01-27

**Authors:** Taylor Jones

**Affiliations:** CulturePoint, LLC., Prince Frederick, New York, NY, United States

**Keywords:** African American English (AAE), lexicalization, social media, language variation and change, morphology, phonology

## Abstract

There are some linguistic forms that may be known to both speakers and linguists, but that occur naturally with such low frequency that traditional sociolinguistic methods do not allow for study. This study investigates one such phenomenon: the grammatical reanalysis of an intensifier in some forms of African American English—from a full phrase *[than a mother(fucker)]* to lexical word (represented here as *dennamug*)—using data gathered from twitter. This paper investigates the relationship between apparent lexicalization and deletion of the comparative morpheme on the preceding adjective. While state-of-the-art traditional corpora contain so few tokens they can be counted on one hand, twitter yields almost 300,000 tokens over a 10 year sample period. This paper uses web scraping of Twitter to gather all plausible orthographic representations of the intensifier, and uses logistic regression to analyze the extent to which markers of lexicalization and reanalysis are associated with a corresponding shift from comparative to bare morphology on the adjective the intensifier modifies, finding that, indeed, degree of apparent lexicalization is strongly associated with bare morphology, suggesting ongoing lexicalization and subsequent reanalysis at the phrase level. This digital approach reveals ongoing grammatical change, with the new intensifier associated with bare, note comparative, adjectives, and that there is seemingly stable variation correlated with the degree to which the intensifier has lexicalized. Orthographic representations of African American English on social media are shown to be a locus of identity construction and grammatical change.

## 1. Introduction

Traditional approaches to quantitative sociolinguistics rely on careful elicitation of naturalistic speech with the goal of counting how many tokens of a particular variant a given speaker uses in a given situation, and relating those to both language internal (structural) constraints and language external (social) constraints on the occurrence of a variant. The investigator may use reading passages, carefully constructed interviews with prompts designed to excite the speaker and lower their self-inhibition [in a Labovian framework, the “sociolinguistic monitor” (Labov et al., 2011); in a psycholinguistics framework, introducing cognitive and emotional interference], or may carefully choose questions to elicit data in a rapid, anonymous survey (“where in this store can I find men's shoes?”). These methods are most effective with tokens that naturally occur with high frequency or that can be easily elicited, for instance deletion (or retention) of postvocalic /r/, or realization of word final ING as either [n] or [ŋ]. However, there are some forms that may be known to both speakers and linguists but which are difficult to elicit and naturally occur so infrequently that traditional sociolinguistic methods do not allow for their study. This is particularly true for African American English, which until recently has primarily been studied by linguists who do not natively speak the variety and who are ethnocultural outsiders (Friedman and Reed, 2020; Hudley et al., 2020), and there is ample evidence that such outsider status can, but does not always, affect data collection in the form of an “interviewer effect” (Rickford and McNair-Knox, 1994; Cukor-Avila and Bailey, 2001). While some features, such as habitual *be* or postvocalic /r/ deletion have been extensively studied, there are other features known to speakers that have received scant or *no* attention in the academic literature (Lanehart, p.c., Smith, p.c., Hall, p.c.). Examples include the associative plural '*nem* (Mufwene, 1998) and the broader change of initial /ð/ to [n] in some phonological contexts, *talkin' 'bout* as a verb of quotation (Cukor-Avila, 2001; Jones, 2016a; Labov, 2018), syntactic change in use of *nigga* (Grieser, 2019; Jones and Hall, 2019; Smith, 2019), and dismissive *bye* among others.

Social media, however, can capture low frequency data that traditional corpora cannot; tokens of interest that may occur a handful of times in a traditional sociolinguistic corpus (e.g., seven instances of third person quotative *talkin' 'bout* and 23 tokens of associative '*nem* in the Corpus of Regional African American Language, Kendall and Farrington 2020) occur hundreds of thousands of times on social media (Jones, 2015). The format is inherently informal (Han and Baldwin, 2011; van Halteren and Oostdijk, 2012; Eisenstein, 2013b), people write for their social networks (Eisenstein, 2013a; Doyle, 2014; Eisenstein et al., 2014; Yuan et al., 2016), and unconventional spellings that pose challenges for traditional NLP applications nevertheless provide rich linguistic information as people engage in identity construction—often through intentionally representing their accents and pronunciation through innovative orthography (Jones, 2016c). People also navigate linguistic taboos orthographically: as Smith (2019) notes, “most white Facebookers (and a few blacks) variably spelled nigga as n***a, nga, ninja, nucca, and nicca, betraying some degree of awareness of the word's taboo status in wider social circles.” The usefulness of social media data for investigating low-frequency forms, especially lexical items, is well established (see, e.g., Grieve et al., 2017, 2018). One largely unexplored avenue of linguistic investigation, however, pursued here, is the use of social media as a window into rebracketing, reanalysis, and syntactic change (Eisenstein, 2015; Jones, 2015; Bleaman, 2020; Jones, 2016a,b,c; Austen, 2017; Jones and Hall, 2019).

The object of study of this paper is the previously undescribed syntactic change, from the complement clause “than a mother(fucker)” to the individual lexical item generally pronounced [dɪnəmʌ:] (rendered here as *dennamug*) in a vernacular register of African American English. I will refer to this as “intensifier *dennamug*” in what follows. This shift is frequently accompanied by *absence* of comparative morphology: a grammatical shift that is indicative of ongoing reanalysis beyond just phonetic reduction, and which is the focus of this paper (1):

(1) a. It's cold*-er* than a motherfucker
b. It's cold-∅ *dennamug*

There is a small number of counterintuitive exceptions to this generalization, discussed in further detail in Section 4 below (2).

(2) Them 8's and Barkley's availabl**er** dennamug...

Intensifier *dennamug* is rare compared to other lexical items, unstudied, and provides a window into linguistic variation and change in AAE outside of the well-described domain of tense, aspect, and mood. Given the recency of study of AAE, and the focus in sociolinguistics on a handful of topics within the study of AAE (describing the tense/aspect/mood system, status with regards to the creole continuum, the relationship between AAE phonology and literacy in the standard language), not much is known about linguistic variation and change in contemporary AAE as relates to lexicalization, reanalysis, and change at the intersection of phonetics, phonology, and syntax.

Intensifier *dennamug* is evidently the result of a number of different, interrelated linguistic processes: it is an intensifier phrase combined with taboo avoidance, understudied AAE phonology, and competing solutions to the problem posed by phonetic ambiguity. Writing on social media requires authors to derive solutions to the orthography problem posed by standard English orthography's inability to capture some aspects of AAE phonology, and this provides a potential window into the etymological transparency (or opacity) of the intensifier.

The confluence of factors prior to writing is the result of phonetic ambiguity that feeds phonetic reanalysis, which in turn feeds syntactic ambiguity that feeds syntactic reanalysis. In other words, the phrase *than a muh(fucka)* is the origin of a single lexical item, with multiple phonological representations in the speech community, that has very different syntactic properties than its origin—the starting point is a full comparative phrase, and the most advanced syntactic change is a lexical item that modifies a bare (i.e., not comparative) adjective.^1^

The present paper seeks to understand the pathway, and more importantly, the *degree* of lexicalization and reanalysis of *dennamug* using all instances of the term on twitter in a roughly 10 year period that are consistent with reasonable orthographic representations of AAE phonology. Here, lexicalization is the degree to which *than a mother* has been from a phrase to a lexical item (e.g., *dennamug*), and reanalysis describes the extent to which it is now treated as a lexical intensifier (which therefore no longer requires comparative morphology on the adjective it modifies). The first sections of the paper describe the phenomenon under investigation, and the subsequent sections investigate the degree of lexicalization and reanalysis using quantitative methods drawing on a corpus of tweets specifically gathered to investigate this topic. Twitter is a mechanism, albeit an imperfect one, for the study of *dennamug* because, despite its low frequency in conversation and the difficulty eliciting it in a traditional sociolinguistic interview setting, there are hundreds of thousands of tokens, and the written format forces speakers to choose whether they write the intensifier as a single word or as a phrase, what the phonological components of the intensifier are, and whether it is accompanied by comparative morphology. Moreover, traditional sociolinguistic corpora are not viable for the present study, as there are no tokens of *dennamug* present in the Corpus of Regional African American Language (CORAAL, Kendall and Farrington, 2020) or Corpus of Contemporary American English (COCA, Davies, 2008), to take two well-respected examples, and only handful of tokens of *than a motherfucker* in CORAAL and 36 such tokens in COCA.^2^ Moreover, in writing, there is no “in between,” as there is in fast casual speech—authors are forced to make choices about how to represent their language that do not allow for ambiguity. The process of reanalysis resulting in intensifier *dennamug* is therefore a perfect illustration of the value of novel computational approaches to sociolinguistics, using social media data, in an area where traditional sociolinguistic methods fail.

Before discussing the materials and methods, it is necessary to discuss lexicalization, intensifiers, comparative phrases, AAE phonology, and taboo avoidance, and to further describe the phenomenon under investigation, as despite the fact that its use is widespread it is nevertheless previously unattested in the academic literature on African American English.^3^ I will treat these in turn in the following sections. With this foundation, I can then return to materials and methods for the present study, which focuses on the extent to which a semantic shift has occurred following fusion and coalescence (Section 1.1), as evidenced by a change in obligatory morphological marking on the adjective *than a mother* ~ *dennamug* modifies.

### 1.1. Lexicalization

Lexicalization, following Brinton and Traugott (2005), is “the process by which new items that are considered ‘lexical'...come into being.” Lexicalization is often contrasted with “grammaticalization,” which refers both to a linguistic phenomenon and field of study (Hopper and Traugott, 2003). The field occupies itself with the “part of the study of language change that is concerned with [...] how lexical items and constructions come [...] to serve grammatical functions or how grammatical items develop new grammatical functions,” and within the field the “steps whereby particular items become more grammatical through time” is referred to as *grammaticalization* (Hopper and Traugott, 2003, pp. 1–2). The distinction between lexicalization and grammaticalization is not always clear, especially in the domain relevant to *dennamug*, in which processes of fusion result in decreased compositionality (Brinton and Traugott, 2005). Indeed, examples of fusion and coalescence, to be defined below, have been treated either as lexicalization or grammaticalization by various researchers, including phrases that have become fixed (e.g., *today* < OE to + dæge “at day-DAT”), derivational affixes derived from roots in compounds, some fixed phrases, multiword verbs, composite predicates or complex verbs (e.g., “lose sight of,” “take action,” “make use of”), and phrase discourse markers (e.g., “I mean”) (Brinton and Traugott, 2005, p. 63–67). Following Wischer (2000), the present study treats the development of *dennamug* as an instance of lexicalization, rather than grammaticalization, because as boundaries and syntactic structure are lost, a specific semantic component is added, rather than semantic components being lost with categorical or operational meaning foregrounded (Wischer, 2000, p. 364–365).

While a broad range of phenomena contribute to lexical innovation, including compounding, derivation, conversion clipping, blending, back formation, and initialisms, among others, the most relevant aspects of lexicalization to the present study are those that relate to reanalysis and change over time, namely univerbation, demorphologization, and idiomaticization. Univerbation is the “unification…of a syntactic phrase or construction into a single word” (Brinton and Traugott, 2005, p. 48–51). A subset of univerbation, sometimes called “delocutivity” (Benveniste, 1971), obtains when an entire phrase is transformed into “a more or less complex word expressing a contiguous concept,” (Blank, 2008, p. 1602, 1604), as in Italian *non so che* “I don't know what” > *nonsoche* “something that is difficult to explain” Spanish *vuestra merced* “your honour” > *usted* “you (formal)” and English *goodbye* from *God be with you*. Some argue that while rare, these are exemplars of lexicalization because they are not just fusion—the obligatory collocation of previously separable material—but also of conversion, in which an item shifts from one category to another. In the case of *dennamug*, the most extreme forms are fully univerbated, and have gone from a comparative phrase to a lexical adverb (Blank, 2008).

Demorphologization describes a process “whereby a morpheme loses (most of) its grammatical-semantic contribution to the word and becomes an indistinguishable part of the construction of the word, while retaining part of its original phonological substance.” (Brinton and Traugott, 2005, p. 52). Indeed, we find demorphologization in *dennamug*, as AAE speakers who use it, and authors in these data, are frequently unaware of any connection to mother and disagree about whether the last syllable is *mub, mud*, or *mug* (see example 6 below).

Lastly, idiomaticization is the extent to which a construction is more idiom-like. What exactly this means in practice is a matter of debate, however Brinton and Traugott (2005) characterize it as comprising three components:

1. **Semantic opacity or noncompositionality**: it is impossible to deduce the meaning of *shoot the breeze* from “shoot” + “the” + “breeze.”
2. **Grammatical deficiency**: an idiom does not permit the syntactic variability characteristic of free combinations such as passive(*the breeze was shot) negation (?*didn't shoot the breeze*), internal modification (*shoot a strong breeze*, *shoot breezes, *shoot some breeze), or topicalization (*the breeze he shot).
3. **Lack of substitutability**: synonymous lexical items cannot be substituted (*shoot the wind, *fire at the breeze), nor can items be reversed or deleted.

Relevant intensifiers in informal AAE span a full spectrum between clearly non-idiomaitic, compositional and substitutable (*than a mother* ~ *than a bitch*), through moderate univerbation and demorphologization while still exhibiting some level of compositionality and substitutability (*danna muv* ~ *danna bish*), to fully univerbated, demorphologized, and idiomaticized (*dennamug*). The best evidence for reanalysis is not just univerbation, demorphologization, and idiomaticization, but subsequent changes elsewhere in the clause or sentence. As will be shown below, the greater degree to which *dennamug* exhibits these characteristics of lexicalization, the greater the likelihood that the adjective *dennamug* modifies appears as a bare adjective, without comparative morphology, because *dennamug* is functioning differently grammatically than the comparative phrase *than a mother*. Before demonstrating this, it is necessary to discuss the morphosyntax of intensifiers (Section 1.2) comparative phrases (Section 1.3) and the interactions between relevant AAE phonology (Section 1.4) and taboo avoidance (Section 1.5).

### 1.2. Intensifiers

Intensifiers are words or phrases that do not modify the propositional meaning of a clause, but add force. They are more or less semantically vacuous, although the degree to which they are more or less depends on the intensifier and context. Intensifiers modifying adjectives come in two types, depending on the adjectives they modify: attributive and predicative (Tagliamonte, 2012).

Attributive intensifiers modify attributive adjectives and can precede or follow the adjective they modify (3):

(3) a. a cold *ass* day
b. *really* cold day

Predicative intensifiers modify predicate adjectives:

(4) a. she's *so* fine
b. she's *really* sweet (Wilson and Gordie, 1957)

Some intensifiers, like *really*, can serve as both attributive and predicate intensifiers, some, like *ass* can only serve as attributive intensifiers, and some, like *deadass* can only serve as predicate intensifiers. Note that *-ass* originates in African American English (Spears, 1998; Collins et al., 2008; Miller, 2017), and that *deadass* is the result of a number of steps of reanalysis: *I'm serious* > *I'm dead serious* > *I'm dead ass serious* > *I'm deadass* > *deadass* + adjective (e.g., *I'm deadass hungry*) > *deadass* + predicate (e.g., “Cuomo is deadass trying to kill us all” apropos of restaurant reopenings in New York during the COVID-19 pandemic).^4^ Note also that *deadass* is the result of grammatical reanalysis of an earlier form, itself the result of reanalysis.

Some intensifiers have historically come from comparisons that lose semantic force, for instance, *pitch* “extremely.” Originally a comparison referring to the black resin used to caulk sailing vessels called *pitch*, as in *as black as pitch* or *pitch black, pitch* now modifies other verbs of perception, as in *pitch quiet* and *pitch silent* (5).

(5) Our old neighborhood was perfect. It was pitch quiet.^5^

A similar case is *as hell*, which no longer draws a comparison to a specific conception of the afterlife, as in *pleasant as hell* “very pleasant.”

The object of study in the present paper is a predicative intensifier (although more will be said about this characterization in Section 4), derived from a comparative phrase. In the next section I discuss comparatives, as reduction of comparative morphology is one form of evidence that *dennamug* is the result of lexicalization and grammatical reanalysis.

### 1.3. Comparatives

Comparatives are similarly complex, and vary across multiple parameters including whether they are bound or periphrastic, clausal or phrasal, and whether they express equality or inequality of degree. English has both bound and periphrastic comparatives (also called synthetic and analytic), hypothesized to be sensitive to the number of syllables the adjective comprises, so bound comparatives are preferred for monosyllables (*easy* + *-er*), and adjectives with more than two syllables almost always occur with a periphrastic comparative (e.g., *more intelligent*, compare to *intelligenter* Jespersen, 1949; Cygan, 1975; Bauer, 1994; Leech and Culpepper, 1997; Lindquist, 2000; Enzinna, 2017). Clausal comparatives take a clausal complement (*Mary is taller than Susan*) whereas phrasal comparatives do not require a comparative clause and may instead use case marking, for instance, the adessive case in Hungarian (Backsai-Atkari, 2014, p. 4). The latter does not occur in English, and therefore will not be discussed further here. Comparatives can express equality (*He's* ***as*** *dumb* ***as*** *a brick*) or inequality (*He's dumb****er than*** *a brick, He's* ***less*** *intelligent* ***than*** *a bag of hair*).

**Figure 1:**
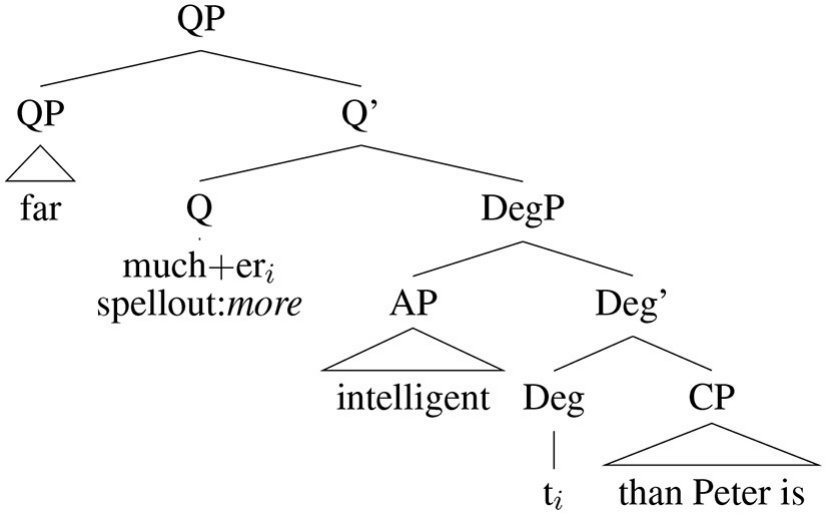
Comparative structure after Backsai-Atkari.

The exact syntactic structure of comparatives has been a matter of lively debate since at least the 1970s, with various structures proposed by Bresnan (1973), Izvorski (1995), Corver (1997), Lechner (1999), Lechner (2004) and Backsai-Atkari (2014), among others. The latter, relying on an analysis that makes use of both Quantifier and Degree Phrases, is assumed here (Figure 1). This is important, because grammaticalization is the result of both rebracketing and reanalysis. Not only does this reanalysis of *than a mother* dramatically change the assumed syntactic categories of its component parts, it also results in an unusual constituent order: an adverb following the adjective it modifies. The important point to note about Backsai-Atkari's proposed structure is that the comparatives *-er* and *more*, while occuring in different syntactic positions, are performing the same function, and that what follows is a clausal complement. The rebracketing and reanalysis of *than a mo(ther)* > *dennamug* is made easier by the fact that AAE phonology is not well served by standard English spelling conventions, and that many expressions and words in AAE are not described in any style guide or dictionary, leaving it to the speakers themselves to determine how to map sounds to spelling. The next section discusses relevant AAE phonology and the following discusses taboo avoidance and deformation relevant to the phonological shape of *than a mo(ther)* as it lexicalizes.

### 1.4. Relevant AAE phonology

An enormous amount has been written about the phonology of AAE, although the focus of much of the sociolinguistic inquiry into AAE has been a relatively small handful of phonological features. Erik Thomas and Guy Bailey summarize the broad strokes in their 1998 and 2015 papers on the subject (Bailey and Thomas, 1998; Thomas and Bailey, 2015), however regional variation in AAE phonology is understudied, and there are understudied phonological features relevant here. Well known variables associated with AAE inlcude variation in - ING, postvocalic /r/ vocalization and deletion, postvocalic /l/ vocalization and deletion, and so called TH-stopping and TH-fronting. Less known and under-researched phonological variables include stop devoicing, debuccalization, and deletion (Farrington, 2018), vowel nasalization, and postocalic /v/ deletion (mentioned in Thomas, 2007 and Jones, 2016b).

There are multiple possible pathways from *mother* to [mʌ:] in AAE, and indeed we find that other words are subject to the same processes (cf. *brother* > *brer* ~ *bruh* [brʌ:]). The word *motherfucker* itself was subject to grammaticalization: it has undergone semantic broadening from an epithet suggesting taboo sexual relations to an individual lexeme that serves as an purpose exclamation, and it is in this context that the first word has undergone reduction. It is often rendered *muhfugga* in writing on social media, reflecting actual pronunciation, attested as early as 1995^6^:

Smokey: “You know what they say, the older the berry, the sweeter the juice.”

Craig: “n—, it's the *blacker* the berry.”

Smokey: “Yeah, well, she blacker than a motherfucker [dɛn ə ^|^mʌfəkə], too.”

(Gray, 1995)

Perhaps interestingly, the earlier widely available recordings of the word *motherfucker* in AAE, as in Richard Pryor's stand up comedy from 20 years prior, are also reduced, but not to the same extent. They also co-occur with a possibly bare adjectives:

I don't blame 'em. Be in a cave two thousand year that'll make you *mad(der) than a motherfucker* [mæd^(ə)^ nə mʌ:v.fʌkə], won't it?

(Richard Pryor, “Mudbone Goes to Hollywood” at the Pryor, 1976)

The process that changes the initial phoneme in words like “than” and “them” to [n], and postvocalic /r/ deletion, along with prosodic factors mean that it is difficult to state with certainty whether Pryor's grammar includes bare adjectives in comparative constructions (and a short epenthetic schwa appears because nasal plosion is not an option in a /dn/ sequence), without further research.

Possible pathways of phonological change leading to [mʌ:] include:

1. /r/ vocalization and deletion > TH-stopping > elision of schwa > postvocalic stop deletion
2. /r/ vocalization and deletion > TH-stopping > elision of schwa > postvocalic /v/ deletion
3. /r/ vocalization and deletion > TH-stopping > elision of schwa > voicing assimilation on postvocalic /v/ preceding onset /f/

Regardless the specific phonological pathway, the result is that *motherfucker* is pronounced, and frequently written, as *muhfucka* or *muhfugga*, and it is this pronunciation that is the starting point for *dennamug*.

The crucial factor here is that the surface phonology of some varieties of AAE allow lax vowels in open syllables, so seemingly un-checked wedge occurs in words like [mʌ:] “mother,” [brʌ:] “brother,” and [lʌ:] “love,” but that these are instances of what Farrington (2018) calls *incomplete neutralization* (in his case, discussing apparent deletion of word final coronal stops, whose voicing specification are still recoverable by vowel length). In this case, a closing consonant is implied, and people writing on social media are compelled to choose one of either < d>, < g>, < v>, < b>, or the generic < h> to avoid readers imagining the unwanted pronunciation /mu/. Some of these forms are more suggestive of truncation (< muh>) or regional AAE phonological processes (< muv>) and others are more phonologically opaque (< mub>, < mud>, < mug>). If speakers do not hear or produce a word final consonant, but know that the surface string has a phonologically illicit long lax vowel, then they can infer that there is a closing voiced consonant, but may be unsure what the precise nature of the consonant is. Perhaps unsurprisingly, there are metalinguistic discussions about which spelling of *dennamug* is “correct”:

(6) a. Dennamug or Dennamud ?^7^
b. Das no typo tho. “than a mub” is a southern saying.^8^
c. yes it is . It a typo cuz its *mug (in response to 6b).^9^

Surprisingly, the responses all indicate ... < mug> is “correct” and none claim *than a mother* is technically correct. While it is also theoretically possible that the /f/ in /mʌfʌɡə/ underwent lenition, and the final schwa underwent apocope, resulting in the change /mʌfʌɡə/→mʌ:ɡə→mʌ:ɡ→mʌ: as a third possible pathway, there is no literature on AAE phonology that would support such a change (i.e., the deletion of an intervocalic fricative when the vowels on either side are the same), and no comparable examples, to my knowledge.^10^ Instead, the last element, to which we now turn, is taboo avoidance.

### 1.5. Taboo avoidance

Taboo avoidance is cross lingusitically common and takes many forms. Different languages may treat different classes of words as taboo, so for instance *ukuhlonipa* “politeness” in the Nguni languages requires deformation of phonemes or syllables related to the names of family by marriage, resulting in a rich set of synonyms. In most varieties of English, the words subject to taboo avoidance are scatological, sexual, and religious in nature (Allan and Burridge, 2006). One form of avoidance is taboo deformation, which can take many forms: minced oaths (*god damn it* > *gosh darn it*), rhyme (*bloody* > *ruddy*), metrical substitutions (*shut the fuck up* > *shut the front door*, or *motherfucker!* > *mother father!*), deletion, and acronyms (*as fuck* > *A. F*. > *ayeff* ).

*Motherfucker* is a taboo word, even thought it may be somewhat meliorated in some informal varieties of AAE (see Spears 1998 and Jones and Hall 2019 for other examples of “so-called obscenity”). While there are multiple strategies for avoiding the taboo, such as the metrical substitution *mother father!*, the strategy relevant here is simply beginning the taboo word, and not finishing it. That is, deletion of most of the phonological material: *mother*fucker. As an expression like *hungrier than a mother* is itself subjected to this treadmill, the result it *than a mo**ther*.

**Figure 2:**
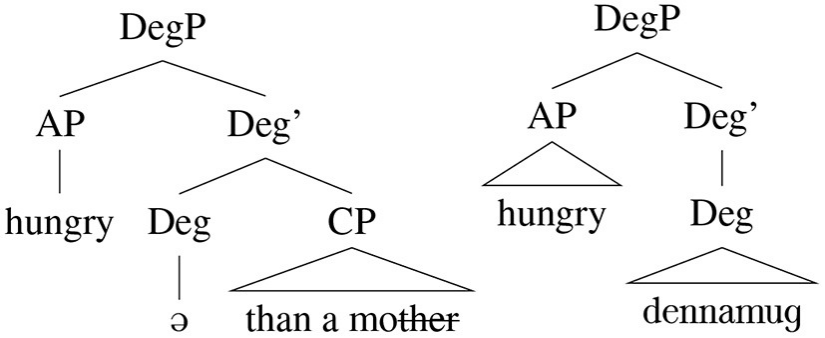
Syntactic reanalysis.

As discussed in Section 1.1, one view of lexicalization relies on reanalysis, and by this view true lexicalization requires rebracketing and a change in the assumed hierarchical structure of a phrase. The combination of a phonological reduction of *mother* in *motherfucker* ([^|^mʌ:fʌɡə]) and taboo avoidance creates the perfect conditions for reanalysis, where the syntactically complex [dɪn [ə [mʌ:]]] is reanalyzed as a single word (Figure 2).

Furthermore, widespread postvocalic /r/ deletion in AAE means that the comparative morpheme *-er* may be realized as a schwa only, and one or two syllable adjectives may contribute to a prosodic pressure toward further reducing the schwa, especially when the following intensifier is not strictly recoverable as a comparative phrase, and especially in fast speech (see, e.g., Davidson, 2006 on pretonic schwa deletion, and compare against AAE *bednot* “better not”):

(7) a. $\bar{\text{fat}}\;\overset{˘}{\text{er}}\;\bar{\text{than}}\;\overset{˘}{\text{a}}\;\bar{\text{mug}}$
b. $\bar{\text{hap}}\;\overset{˘}{\text{py}}\;\overset{˘}{\text{er}}\;\bar{\text{than}}\;\overset{˘}{\text{a}}\;\bar{\text{mug}}$

In the next section, evidence for metalinguistic discussion of *dennamug* among AAE speakers is adduced as further evidence for a cline of lexicalizing forms. In Section 2, I turn to the materials and methods for the present study, investigating the extent to which there is quantitative evidence for rebracketing and reanalysis.

### 1.6. Metalinguistic awareness as evidence for reanalysis

Further evidence for possible lexicalization comes from how speakers themselves discuss the language. Beyond exchanges on twitter like that in example 6, above, there is evidence that some AAE speakers believe *dennamug* to be a lexical item and not a comparative clause. Urban dictionary has an entry from 2006 for *than-a-mug* with the definition “To the extreme of something's current state,” and an entry from 2003 for *dennamug* with the definition “hella, a lot, very much.”^11^,^12^ Absent is any reference to the expression “than a motherfucker.” The latter definition predates the launch of twitter by 3 years, demonstrating that *dennamug* cannot be merely an orthographic meme on social media. Evidence of speaker perceptions are not limited to written attestations, either: for instance, in 2013, YouTube personality Kevin Fredericks (known as Kev On Stage) made a video of “Black Folks Slang” in which explains *dennamug* (which he spells on screen as < dennamug>) is “the measure of unit of something that is something else” and elaborates that “whatever *dennamug* is, that unit measure, you've gotta be doing *more* than that.”^13^ While this is not necessarily how a linguist would phrase it, it is nevertheless clear that he is describing an intensifier.^14^ He pronounces a word final /g/ in citation form, but then provides six example sentences of his own with no closing /g/.^15^ At no point does he make reference to the comparative phrase *than a mother*. Of the six examples of his own he provides, five had comparative morphology, and one did not, of the five examples he provides from others, none had comparative morphology.

It should be clear from the above that *dennamug* is (1) a phenomenon in spoken AAE that (2) speakers expect others to understand, and as such, it is widespread (occuring in movies, radio, television, stand up comedy, YouTube videos, get-out-the-vote ads,^16^ political rallies with former presidents,^17^ etc.), and (3) its origins as a comparative phrase are opaque to some speakers, who now perceive it to be a single word, and who no longer consistently use it with comparative morphology. It is historically related to, but distinct from, the comparative phrase *than a motherfucker*. Despite being widespread, it is just the type of phenomenon that is difficult or impossible to study using traditional sociolinguistic methods and corpora. However, this is precisely a situation in which new computational methods, in this case as simple as web scraping, allow for sociolinguistic insights. Lexicalization is generally understood to be a slow process that unfolds over time, and one for which both older and newer forms overlap. Moreover, reanalysis does not necessarily entail immediate change in surface manifestations (Langacker, 1977), but such change is a strong piece of evidence for reanalysis. In the next section, I discuss materials and methods used to investigate the extent to which reanalysis has occurred, using absence of the comparative morpheme *-er*—obligatory in comparative phrases—as an indicator of reanalysis and lexicalization.

## 2. Materials and methods

For the present study, I gathered all tokens of all spellings of *dennamug* on twitter in the 10 year period from 2007 to 2017 that are consistent with reasonable orthographic representations of AAE phonology. To do so, I accounted for TH-stopping by searching for both an initial < th> and initial < d>; I accounted for raising of /æ/ and the pin-pen merger by searching for < a>, < e> and < i>; I accounted for one-, two-, and three-word spellings and accompanying duplication of orthographic < n> in single and two-word spellings (as in < dinna muh>); and I searched for word final < d>, < g>, < v>, and < h>. I did not initially search for word final < b>; however, I later gathered all 143 tokens manually. While it is possible there are unaccounted for spellings, they are so rare as to be irrelevant to the analysis here (in fact, many of the tokens generated by this algorithm returned one or no tweets). I used a shell script to generate all possible spellings meeting the above criteria and to make individual calls to the now deprecated get-tweet script in Python.^18^ The tokens sought were thus any that matched:


(th|d) (a|e|i)n+\\s?a\\s?mu (b|d|g|v|h)

The resulting data set comprised 294,364 tweets, plus another 143 observations with a word final < b>, for a total of 294,507 tweets. After eliminating false positives (e.g., *she's uglier than a mud fence*, or *nothing better than a mug of hot chocolate*), eliminating tweets that included some spelling of *fucker* immediately following the token, and eliminating false positives in other languages,^19^ 264,816 tweets remained.

Examples of true positives include:

- I know terence blanchard bouta be playing that trumpet louder than a mug lol.
- Back sore dan a mug from rehearsal i could use a back rub.
- Man one of my friends is long winded den a mug dawg she can talk yo fuckin ear off.
- Pook auntie funny den a mud.^20^

Data gathered included tweet ID, username, tweet text, date, time, retweets, likes, geolocation (where applicable), mentions, hashtags, and permalink. I created variables for which token was contained in the tweet (e.g., *danna muv*), preceding adjective, and whether comparative morphology was present or absent. User profile pictures were also collected. While, in principle, these could be used to code gender (or more accurately, gender presentation), approximate age, and (not self-identified) “race,” those were not coded for in the present analysis and remain an area for future inquiry, however, there is no *a priori* reason to think gender and age are relevant to use of *dennamug* and race is only relevant insofar as it is a highly correlated but imperfect proxy for use of AAE. ^21^ Nevertheless, visual inspection of the profile picture data suggest that the subjects are not imbalanced by gender, and are overwhelmingly Black and American. Unfortunately, visually inspecting and hand coding for apparent age and gender presentation was unfeasible. Such inspection and coding would also be fraught with methodological and ethical challenges, including but not limited to own-age and own-race biases on the part of the researcher and image misattribution (for instance, when a twitter user's profile picture is of a relative, celebrity, or other person who is not the author). The rest of the language in the tweets exhibits both orthographic representations of AAE phonology (pin-pen merger, coda cluster simplification, TH-stopping, TH-stopping, etc.), AAE morphosyntax (e.g., habitual *be*, stressed *been*, preterite *had*, copula deletion, etc.), and AAE lexical items that have not yet been borrowed by the white mainstream (e.g., *saditty, bama, ashy, jont, darkskin, geeked, siced*, etc.). I normalized the most common variant spellings, changing word final < a> and < ah> to < er> and normalizing arbitrarily many repeated letters, as in < sleeeeepy> to < sleepy>, but did not normalize other respellings that were not merely lengthening, as in < fye> “fire” or < asapidlier> *ASAPedly-er* “quicker.”^22^

The two users who tweeted the most, NICKNCEJAIGH with 615 tweets and 101THEGREAT with 478 tweets, were marketing their original songs “Harder than a mug” and “Fresher than a muh,” respectively, both of which use the intensifier in the hook. Because the tweet texts, while unique, were using *dennamug* in citation and not actual use, I did not retain tweets from these two authors. Because they shared links to their songs, however, there is further evidence for pronunciation. Despite the orthographic representation, both dramatically phonetically reduced the intensifier, with NICKNCEJAIGH saying [ʃi ɡo: hɑ:də dɪnə^|^mʊ:h] “she go harder *dennamug*,” and 101THEGREAT singing [~ɑ frrɛʃə d~ɪ nə^|^mʌ:h] “I'm fresher *dennamug*.”^23^

**Figure 3:**
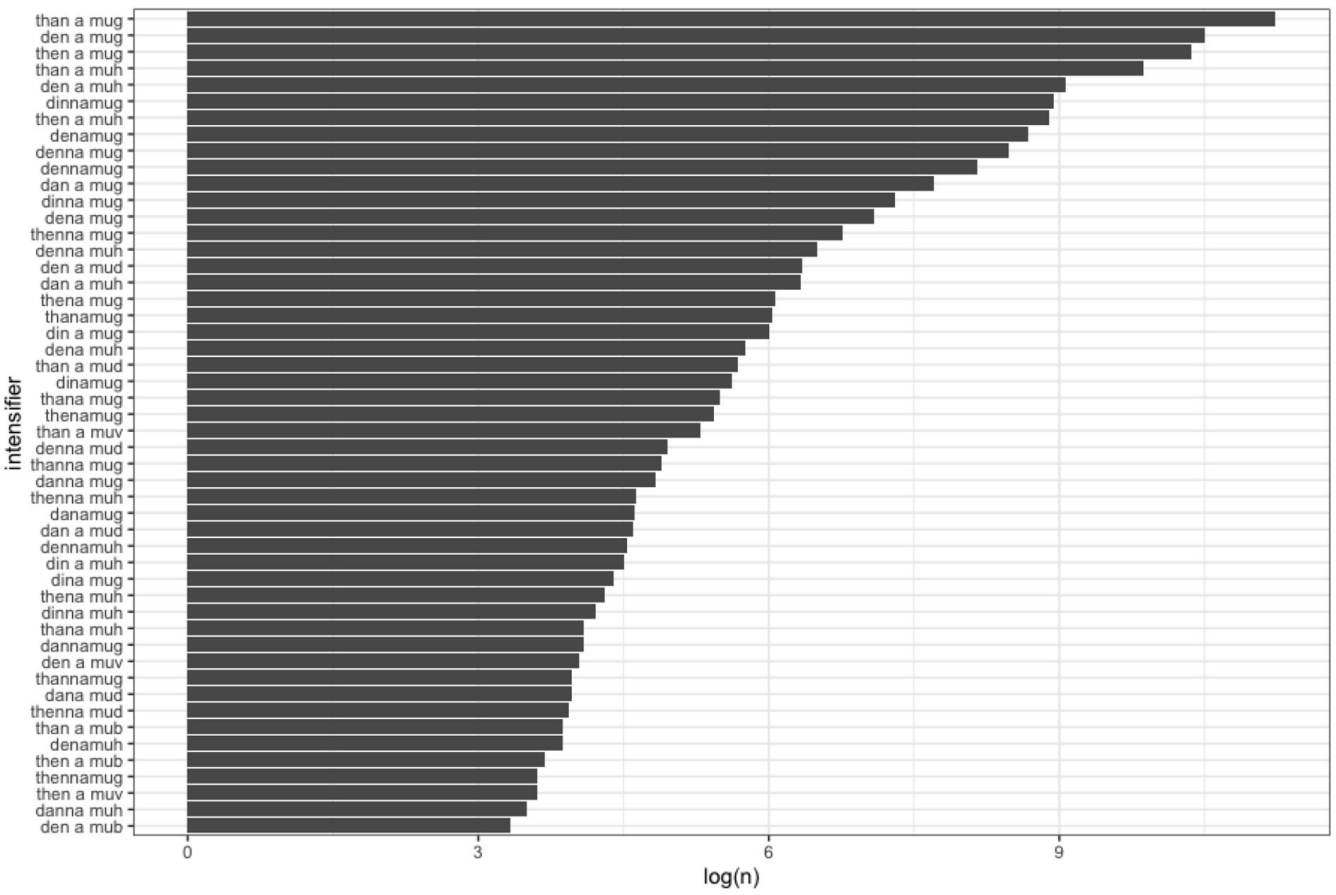
Fifty most common intensifiers (log transformed).

**Figure 4:**
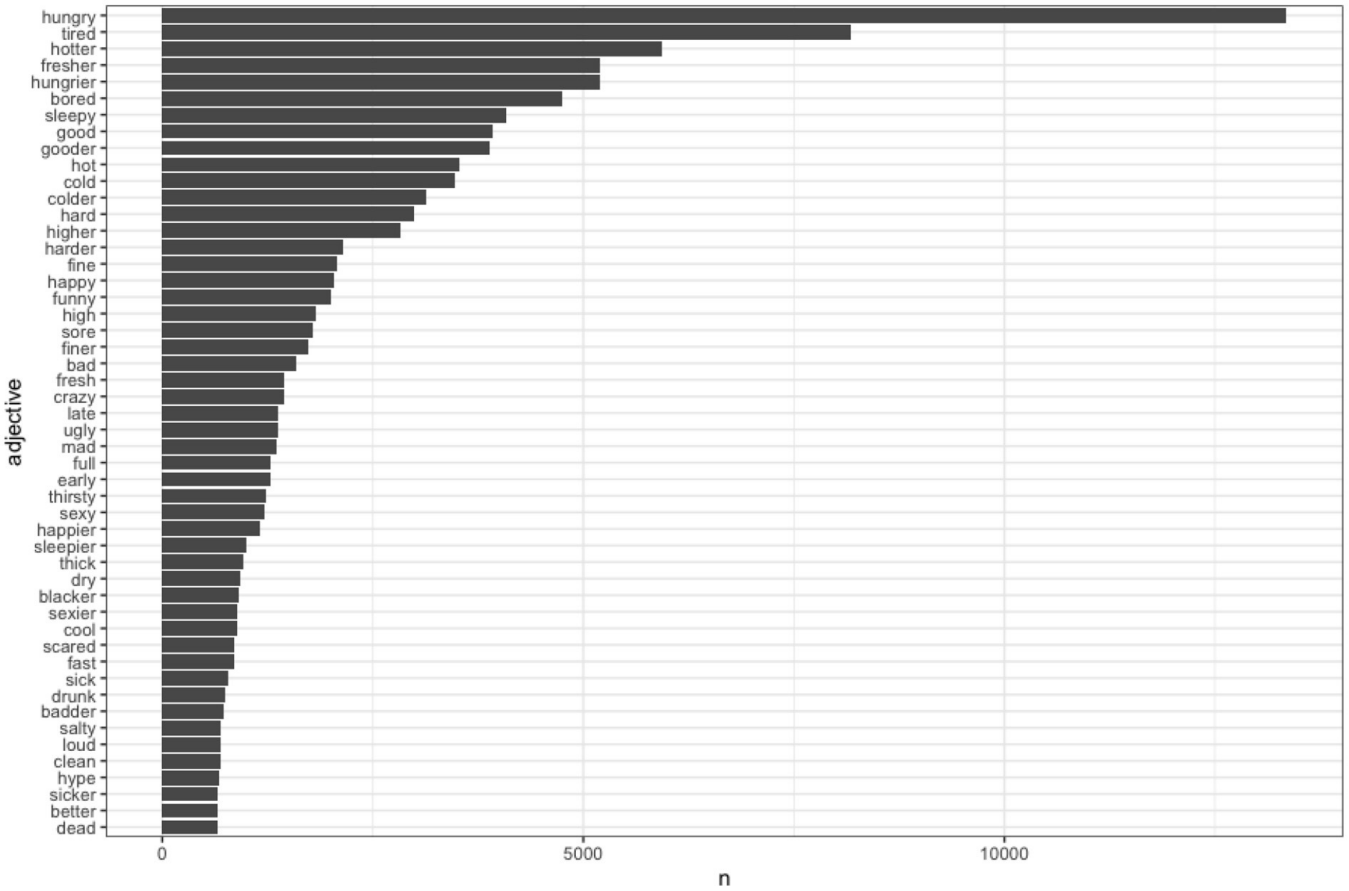
Fifty most common adjectives (normalized spelling).

The fifty most common spellings of intensifier *dennamug* are presented in Figure 3.^24^ Because these follow the expected Zipfian distribution, they are presented log transformed. The fifty most common adjectives, after spelling normalization, are presented in Figure 4.

Of primary interest was the relationship between orthographic indicators of reanalysis as a single, opaque lexical item, and presence or absence of comparative morphology. To investigate this I performed both traditional logistic regression, and mixed effects logistic regression to account for unmeasured author characteristics. The response variable was presence of comparative morphology on the adjective (that is, some orthographic representation of a final *-er*). The predictor variables were the presence or absence of orthographic representation of TH-stopping (initial < th> or < d>); the orthographic representation of initial vowel (with < a> as a reference category); the orthographic representation of the final “closing” consonant (with < g> as a reference category);^25^ complexity (meaning, how many orthographic words); lemma frequency (where *hungry, hungrier*, and *hongryyyyyy* all count as tokens of hungry); whether the lemma was *good* (to account for the now enregistered *gooder dennamug*); and random intercepts in the mixed effects model for username to account for unmeasured author characteristics. The form of the intensifier used was not included, as the first four variables completely and uniquely describe it (for instance, TH-stopping, an initial /e/, complexity of 1, and final consonant /g/ selects *dennamug*), and any model that included it would suffer from severe multicollinearity. Similarly, adjective lemma was not included, as lemma frequency was highly correlated with it.^26^

Because the number of tweets per author followed a power distribution with the vast majority of user IDs associated with only a single tweet, mixed-effects logistic regression with a random term for username was not feasible on the full data set. However, it is possible that unmeasured author characteristics had an effect on rate of comparative morpheme deletion. To overcome this limitation, I performed logistic regression without a random term for username on the full data set, and performed goodness-of-fit tests, then performed logistic regression on the subset of the data comprising authors who tweeted only once, and performed mixed effects logistics regression with a random term for username on a subset of the data that encompassed all users who tweeted *at least* 10 times,^27^ which represented 52,646 observations from 2,442 authors. The results were consistent and robust across multiple specifications of the model.

The distribution of final consonants was heavily skewed (toward < g>), as was the distribution of orthographic complexity (with two spaces heavily preferred, followed by none). Because the final consonants are easily divided into two natural classes (i.e., voiced stops, comprising /b/, /d/, and /g/, and fricatives, comprising /h/ and /v/), the model was run with a binary variable for *fortition*. Similarly, because any reduction in orthographic complexity is a sign of reanalysis, the model was run with a binary variable for *complexity*. This form of the model significantly outperformed others that had five categories for final consonant and three categories for orthographic complexity.

The form of the basic model was therefore:


(1) $$\begin{array}{l} \begin{array}{l} Comparative=\beta_{0}+\beta_{1}THstopping+\beta_{2}Vowel\\ \;+\beta_{3}fortition+\beta_{4}Complexity\\ \;+\beta_{6}LemmaFrequency+\beta_{7}isGood+\epsilon \end{array} \end{array}$$

and for mixed effects logistic regression:


(2) $$\begin{array}{l} \begin{array}{l} Comparative_{ij}=\beta_{0}+\beta_{1}THstopping_{ij}+\beta_{2}Vowel_{ij}\\ \;+\beta_{3}fortition_{ij}+\beta_{4}Complexity_{ij}\\ \;+\beta_{6}LemmaFrequency_{ij}+\beta_{7}isGood_{ij}\\ \;+\beta_{8}Author_{j}+\epsilon_{ij} \end{array} \end{array}$$

Model comparison and post-estimation tests confirmed that these models outperformed similar models that dropped variables included in these models. It also dramatically outperformed models that included *year*, which was not significant in the models that included it, and in some cases caused failure to converge. Vowels other than < a>, final consonants other than < v> and < h>, and reduced orthographic complexity (suggestive of univerbation), were anticipated to be associated with greater comparative deletion. TH-stopping was not expected to be associated with change in comparative morphology, as it is a productive process in AAE phonology. Use of the lemma *good* was expected to be associated with an increase in comparative morphology, because of the enregistered idiom *gooder than..*.. Lemma frequency was likewise expected to be associated with greater use of comparative morphology.

## 3. Results

The results of the logistic regression performed on the full data set are presented in Table 1. All predictor variables are significant at the 0.001 level. The intercept of 0.43 indicates that all things being equal, the probability of encountering comparative morphology on the preceding adjective was only 60.4%. Th-stopping was associated with a small, but significant *increased* probability of encountering comparative morphology on the preceding adjective (see below for discussion). Initial vowels other than < a> were associated with a significant decrease in probability of comparative morphology (33 percentage points for < e> and 28 percentage points for < i>). Fortition of the final consonant, and reduced orthographic complexity were both associated with significant decreases in probability of encountering comparative morphology. Lemma frequency was associated with a small but positive effect—more common words were more likely to exhibit comparative morphology, all things being equal. The word *good* was associated with a significant, positive effect: if the lemma was *good*, it was much more likely for the form of the word to be *gooder*, regardless of the form of the following intensifier (as anticipated). All things being equal, comparative morphology on the adjective was associated with a probability of 0.15 of appearing before a univerbated intensifier with initial /d/, raised first vowel, and a word-final stop (e.g., < dennamug>, < dinnamud>, etc.). That is, univerbation and phonological opacity obscuring the relationship to *than* and *mother* were associated with a dramatic loss of comparative morphology on the adjective.

**Table 1:** Results of logistic regression on the full data set.

	**Estimate**	**SE**	***z* value**	**Pr(>|*z*|)**
(Intercept)	0.43	0.01	37.15	0.00***
thStoppingTRUE	0.19	0.02	12.27	0.00***
Vowel: e	–1.41	0.01	–105.10	0.00***
Vowel: i	–1.17	0.03	–37.19	0.00***
Fortition	–0.82	0.01	–68.59	0.00***
Complexity: reduced	–0.10	0.02	–4.99	0.00***
Lemma frequency	0.05	0.00	9.97	0.00***
Adj: good (TRUE)	1.25	0.02	58.46	0.00***

The *** symbol indicates the significant at the 0.001 level.

Performing logistic regression on the subset of tweets for which the author only tweeted once, the results are similar, and are presented in Table 2. In this subset of the data, th-stopping is no longer a significant predictor. The effect directions are the same, and the magnitudes are approximately the same as in the model on the full data set, except for the effect for orthographic complexity, which is larger by a factor of three.

**Table 2:** Results of logistic regression on the subset of data comprising authors who tweeted once.

	**Estimate**	**SE**	***z* value**	**Pr(>|*z*|)**
(Intercept)	0.86	0.02	49.64	0.00***
thStoppingTRUE	–0.02	0.02	–0.91	0.36
vowele	–1.38	0.02	–72.83	0.00***
voweli	–0.86	0.08	–11.15	0.00***
Fortitionfortition	–0.91	0.02	–49.87	0.00***
complex2reduced	–0.36	0.04	–8.94	0.00***
Scale(lemmaFreq)	0.07	0.01	9.26	0.00***
isGoodTRUE	1.00	0.04	25.54	0.00***

The *** symbol indicates the significant at the 0.001 level.

Finally, the results of mixed-effects logistic regression accounting for unmeasured author characteristics on those who tweeted at least 10 times is presented in Table 3. The intercept is –1.52, compared to 0.86 for those who tweeted once, indicating that all things equal, those who tweeted once were likely to tweet with comparative morphology 70% of the time, whereas those who tweeted 10 or more times were only likely to tweet with comparative morphology on the adjective 18% of the time.

**Table 3:** Results of logistic regression on the subset of data comprising authors who tweeted 10+ times.

	**Effect**	**Group**	**Term**	**Estimate**	**SE**	***z*-value**	***p*-value**
1	Fixed		(Intercept)	–1.52	0.11	–14.15	0.00***
2	Fixed		TH-stopping (TRUE)	0.54	0.08	6.51	0.00***
3	Fixed		Vowel: e	–1.64	0.08	–19.91	0.00***
4	Fixed		Vowel: i	–1.62	0.12	–13.51	0.00***
5	Fixed		Fortition	–0.46	0.09	–4.78	0.00***
6	Fixed		Complexity: reduced	–0.01	0.07	–0.22	0.83
7	Fixed		Lemma frequency	–0.02	0.02	–0.99	0.32
8	Fixed		Adj: good (TRUE)	2.21	0.06	35.23	0.00***
9	Ran_pars	Username	sd__(Intercept)	2.51			

The *** symbol indicates the significant at the 0.001 level.

For this subset of the data, th-stopping is once again significant, and positively associated with the presence of comparative morphology. Raising of the initial vowel was associated with loss of comparative morphology, as was fortition of the closing consonant. Orthographic complexity and lemma frequency were not significant.

Across all of the data, most common form was < than a mug>, with 82,944 tokens. Extrapolating from the coefficients in the first model, the probability of seeing comparative morphology on the adjective for this form was 0.41 or approximately two in five. The forms that are most transparently related to *than a mother*, < than a muv> and < than a muh>, have a probability of 0.61 of appearing with an adjective that exhibits comparative morphology—by far the highest probability of any forms that actually appears in the data. The least transparently related possible forms to the original comparative phrase, (e.g., < dinamub>, < dinnamud>, etc.), were predicted to have a 0.19 probability of appearing with comparative morphology. The form in the data associated with the greatest likelihood of comparative deletion is < thenna mud> which appears 60 times in these data and never with comparative morphology on the adjective. The variable associated with the largest *increase* in likelihood of comparative morphology on the preceding word was whether the preceeding word was a form of *good*, although ironically, this was not due to preference for the word *better*, but rather for *gooder*.

## 4. Discussion

Taken together, these results suggest that when looking at *dennamug* and not *than a mother*, we are not merely looking at creative orthography to represent spoken accent (although there is strong evidence for this in AAE as well; see Jones, 2016c, for a thorough discussion). Rather, we are also looking at evidence of lexicalization and grammatical reanalysis. Almost any nonstandard spelling of *than a m2:* is already likely to show bare morphology on the adjective, but the more additional orthographic evidence of univerbation and demorphologization—spelling the intensifier as fewer than three orthographic words, closing the syllable with an (unpronounced) < b>, < g>, or < d>, changing the initial vowel so the first syllable is no longer transparently *than*—are all associated with greater probability of encountering a bare adjective.^28^ The intercept suggests that all things being equal, the probability of seeing comparative morphology with some form of *dennamug* is already only two in three, which alone is strong evidence for lexicalization and grammatical reanalysis: comparative morphology before a comparative phrase is syntactically obligatory elsewhere in AAE, and with very few exceptions, intensifiers precede the adjective they modify.^29^

In many varieties of AAE, a prenasal /æ/ can be realized as [ɛ] (Jones, 2020).^30^ In the syntactically ambiguous context here, that ash-to-epsilon shift can then feed the pin-pen merger. Not only is this consistent with some of the written forms (e.g., < dinnamug>) and with the common pronunciations, but this is only possible if the initial syllable is no longer clearly “than” to all speakers. Similarly, the above findings are consistent with the hypothesis that *mother* is more recoverable from < muv>, (cf *muv* [mʌvə] “mother” in some varieties of AAE), and less recoverable from the non-word < mub> or from the words < mud> and < mug> which may be associated with lexical interference. Indeed, as noted above, the form most likely to exhibit comparative deletion was < thenna mud>, which is not only no longer transparently *than a mu:*, but is also spelled in such a way that each component invites lexical interference.

It should be noted that, at least on twitter, authors seem to have a high level of awareness that *dennamug* is a non-prestige form (although I'm reluctant to call it non-standard, since many appear to have strong feelings about which spelling is “correct,” and there is a *de facto* emerging standard spelling). *Dennamug* has already become enregistered (Agha, 2003) for some AAE speakers (e.g., Kev On Stage fans). There is an enormous amount of linguistic awareness and playfulness in these data. While the adjectives *dennamug* appears with follow the expected Zipfian distribution, the long tail of lower frequency items has a preponderance of uncommon words from high or academic registers, often in a comparative construction that does not work in a classroom setting: *ostentatious, temperamental, rhetorical, vomitous, delectable, subpar, bowlegged, incognito, belligerent, inebriated, jovial, dejavu*, and *schadenfreude (dinnamug)* among many others. Moreover, authors employ comparative morphology on words that do not ever receive comparative morphology in standard English: *antisocialer, catholicer, beautifuler, startlinger, overrateder, sunburnter, negligenter, tirededer, fadeder* (i.e., “drunker”), and *confuseder*, among others. Most prominent of these is *gooder*, which occurs 3,778 times in these data, and is sufficiently enregistered that there are multiple songs with the name “gooder dennamug.”^31^

**Figure 5:**
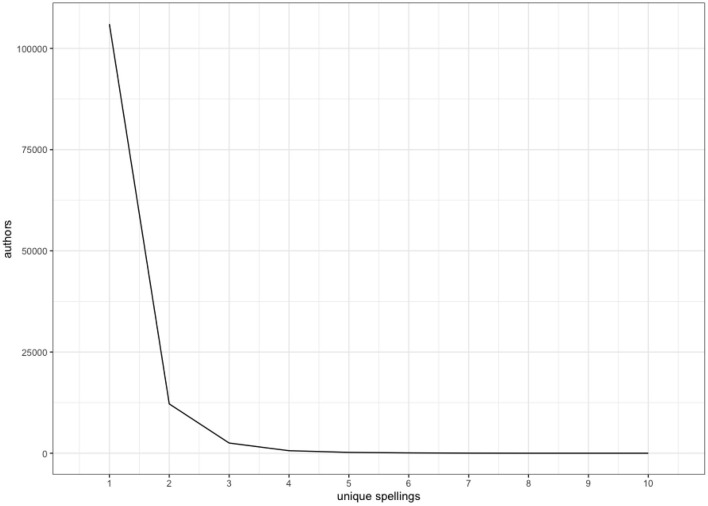
Number of authors by number of unique spellings.

**Figure 6:**
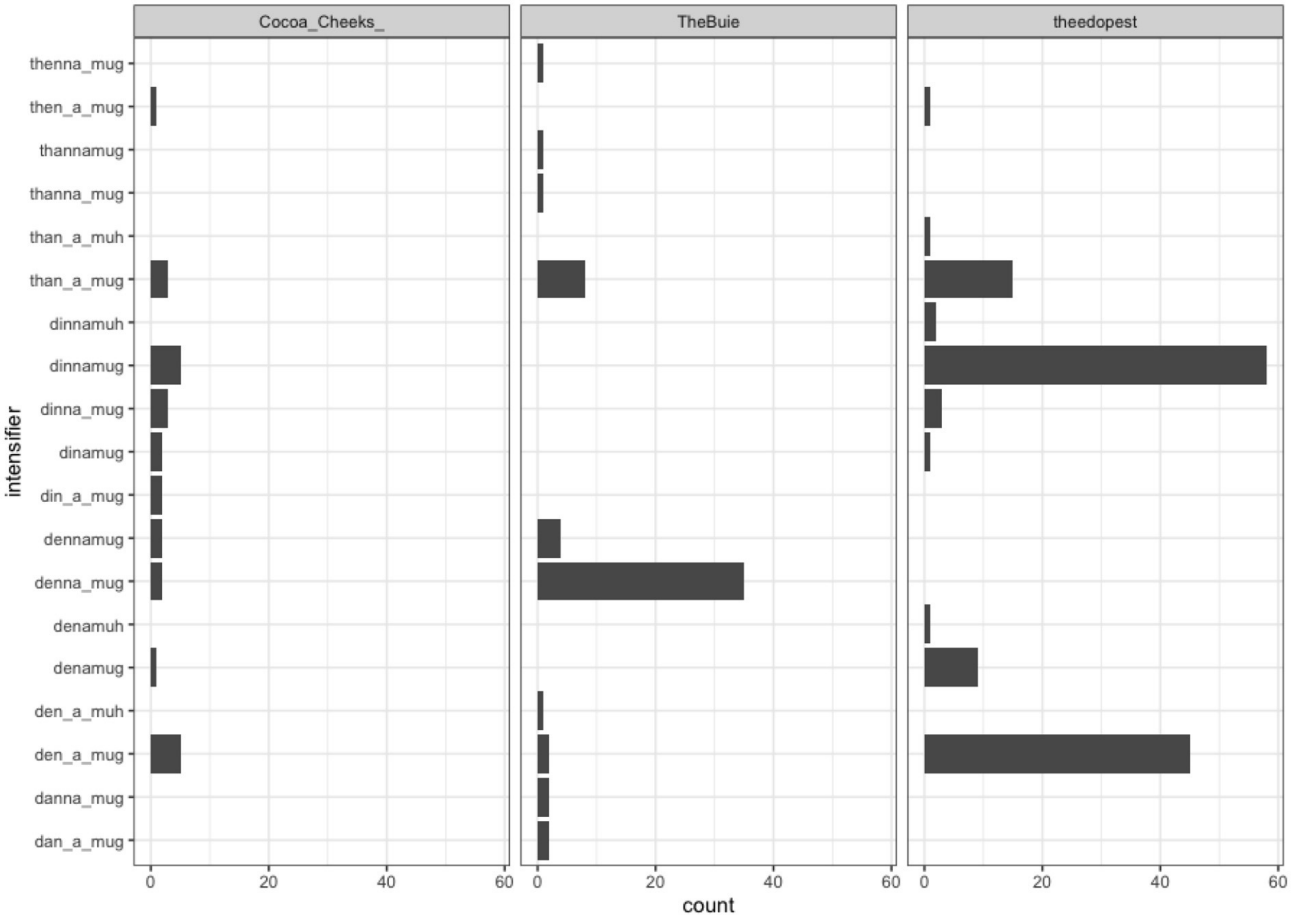
Orthographic choices for authors who used 10 different spellings.

There is also significant intraspeaker variation. Examining the tweets of the 32,547 individual authors who wrote at least two tweets with some form of *dennamug* in them, 16,884 used a single spelling (the most prolific, tweeting < than a mug> 244 times, but even two of the top five most prolific stuck to < dinnamug>, with 98 and 85 such tweets from the fourth and fifth most prolific, respectively. The remaining 15,663 authors who tweeted at least twice using some form of *dennamug* did made use of multiple spellings (Figure 5). There is no apparent temporal pattern, so it appears as though authors are solving the spelling problem posed by *dennamug* on the fly, and re-solving the problem each time. Three of the authors made use of up to ten distinct spellings, and crucially, they were not the same spellings across these authors (Figure 6). There is, therefore, strong evidence of both inte- and intra-speaker variation in terms of how speakers choose to represent *dennamug* orthographically, suggesting that speakers are not always certain how to phonologically or syntactically bracket the expression.

*Dennamug* is also exhibiting even further lexicalization and possible grammaticalization in these data. While it was not feasible to automatically parse part of speech for the full data set as POS taggers are still not at a satisfactory level, with state of the art approaches performing at ~80% on tweets (Jørgensen et al., 2016), manual inspection of the data reveal interesting avenues for future study. Not only does *dennamug* modify adjectives, but it is now able to modify adverbs:

(8) a. She is driving leisurely than a mug
b. I am employed....gainfully then a mug too

It can now modify noun phrases:

(9) a. binary thinking *(dennamug)*
b. false advertising *(dennamug)*
c. foreshadowing *(dennamug)*
d. Power trip *(dennamug)*

It can now modify prepositional phrases:

(10) My accent gonna be outchea dennamug because I'm going to be exhausted^32^

It can now modify verb phrases:

(11) a. Procrastinating dennamug
b. Back when Trick was hot I was illegally dl'ing den a mug^33^
c. That chick is line stepping dennamug
d. I'm laughing dennamug cause I'm sure they gone get rid of your favorite. Trust me
e. Projecting dennamug!!
f. Tweet watching dennamug
g. I be scanning dennamug on a day like this here.
h. Cramping dennamug

And it can even modify entire clauses and sentences:

(12) a. No weapon formed against me shall prosper. Dennamug.
b. “You one of them or one of us?” That was a loaded question dinamug. #ShotsFired

Unfortunately, there are many social factors that the present study cannot disentangle. It is clear that beyond linguistic playfulness, there is an element of Black identity construction at play for many of the authors of these tweets, and authors recruit a variety of AAE features to construct or hint at “Black” *personae* (D'Onofrio, 2020; King, 2021). Many write < fahn> for [fɑ:n] “fine,” representing /ay/-monophthongization, or < fye> for *fire*, capturing postvocalic /r/ deletion. Example 12a is particularly interesting because it is an ironic use of *dennamug*, which relies on the audience finding the humor in juxtaposing sacred and profane, within a Black American Christian context, for comedic effect: The sentence is a reference to the 1996 Fred Hamilton song “No Weapon,” which is itself an adaptation of Isaiah 54:17, likely from the New King James Version translation “No weapon formed against you shall prosper”^34^, and here *dennamug* is replacing the expected affirmation, *amen*. Other low-frequency, difficult-to-study, yet nevertheless attested AAE phenomena abound, for instance, the shift from /t/ to /k/ in initial sCr clusters (see, e.g., Bailey and Thomas, 1998, p. 89):

(13) a. I'm hongrier than a mug in this class. Lord, please give me skrenf [strength]^35^
b. Hot than a mug out here in these skreets... [streets]
d. it's skrowng than a muh [strong]^36^
man that game skressful thanamug [stressful]

There is much future research that could, and should, be done on this subject. One domain for future inquiry is the possibility of age grading (Hockett, 1950; Labov, 1994; Tagliamonte, 2012). One friend of the author asked “are people still saying that?” when told about this study, and indeed, there are suggestions in the data that age grading may be a very real possibility, e.g.:

(14) Just realized I'm too grown to be saying dennamug

Future work could make use of targeted elicitation, and of surveys, to better tease apart social factors related to adoption and use of this form. It is possible that autocorrect plays a role in the strong preference for < mug> and < mud> in the written data. It should be noted that those who chose to write < than a mub> must intentionally override autocorrect. There may also be lexical interference from words like *mug* and *mud*, that a future psycholinguistic study could disentangle. One important question future research should address is the question of whether bare adjectival morphology is the result of lexicalization of *than a mother* or if it preceded and fed that lexicalization. I am unaware of academic literature on bare adjectives in comparative constructions in AAE, however the phenomenon is known by AAE speaking linguists (for instance, Hiram Smith provides the example *she fine than a sumbitch* in a personal correspondence). The temporal ordering of these changes, whether it's bare adjectives → reduction of *than a motherfucker* to *dennamug* or vice versa, will provide important insight into pathways of grammatical change in AAE.

Nevertheless, while there is still much more to tease apart, it is clear from the above that AAE *dennamug* is an intensifier that is the result of ongoing lexicalization. Moreover, this ongoing lexicalization would have been impossible to study just a few years ago, despite being in widespread use among AAE speakers, not just because it is unlikely to appear in more formal written registers,^37^ but also because the burden of proving even the existence of the phenomenon would have been to difficult for linguists using traditional methods, and the volume of data too low for analysis. The new discipline of computational sociolinguistics offers methodological innovations that allows linguists to investigate phenomena, at large scale, that we may have only heard fleetingly in the field. This broadening of methodological horizons entails a broadening of possible linguistic objects of study, and allows us to compile and study corpora of understudied languages (or linguistic phenomena) while simultaneously benefiting from linguistic transcriptions performed by the speakers themselves, rather than linguists, however well-trained. This in turn, can allow for a new window into linguistic variation and change.

## Data availability statement

The datasets presented in this study can be found in online repositories. The names of the repository/repositories and accession number(s) can be found at: https://github.com/TaylorWJones/dennamug.

## Author contributions

The author confirms being the sole contributor of this work and has approved it for publication.

## Footnotes

^1^There is limited evidence, discussed in Section 4, that this lexical item is on its way toward modifying other types of phrases, as well.

^2^Some of these tokens are from non-AAE speakers writing for AAE-speaking characters, as in *The Wire*, others are written and delivered by AAE speakers as in *Friday*, but COCA has the written script, and not what was actually spoken.

^3^This is another reason for using quantitative methods: linguists unfamiliar with the term may doubt its existence; the data for the present analysis and the analysis itself are independently verifiable and do not rely on readers trusting an analysis of an unverifiable data set, as would be the case in traditional ethnographic and sociolinguistic field work.

^4^ https://twitter.com/QueeringPsych/status/1355329178695041025

^5^ https://twitter.com/DomoDash_/status/1344495542882201603

^6^Note that there is a range of pronunciations of the last two syllables as well, from [fʌkə] to [fʌɡə] to [fʌ:].

^7^ https://twitter.com/bydie_ching2x/status/547810865442226176

^8^ https://twitter.com/MoeMyGod/status/229455319460356096

^9^ https://twitter.com/JaCorii_/status/229457265239928833

^10^For instance, in varieties of AAE that front /ð/, as in mʌvə “mother,” there is no attested evidence of such reduction, as in *my mʌ: “my mother.”

^11^ https://www.urbandictionary.com/define.php?term=than-a-mug

^12^ https://www.urbandictionary.com/define.php?term=dennamug

^13^ https://www.youtube.com/watch?v=RRriNPDpmGU

^14^His description implicitly agrees with the last few decades of research on comparative phrases, which situate them in DegP, as well.

^15^This was determined perceptually and with the aid of spectrographic analysis in Praat, which confirmed there is no “velar pinch” consistent with a closing velar consonant.

^16^ https://deadline.com/2022/09/cedric-the-entertainer-hershel-walker-video-1235120898/

^17^ https://news.yahoo.com/woman-tells-obama-hes-finer-211110070.html

^18^Twitter has historically been antagonistic to those who would scrape data, and has changed the way tweets are represented in a browser to render this script and others like it ineffective. The primary mechanism was to automate endless browser scrolling (and refreshing) and parse the html.

^19^After removing other character sets, I used automatic language detection with cld3 to eliminate non-English tweets (mostly Indonesian). I manually checked these, as language detection is unreliable with AAE (Blodgett et al., 2016, 2020). For instance, of over 400 tweets categorized as Spanish, only 11 were actually in Spanish. The rest were evidently characterized as Spanish due to the presence of English discourse marker *yo*, or mention of place names in the Southwest (e.g., El Paso).

^20^“Pook's auntie is extremely funny.”

^21^Within a Labovian framework it may be reasonable to hypothesize that if use of *dennamug* isa change in progress, that it may be driven by young women, however these data do not permit us to investigate this possibility. Anecdotally, this author hears *dennamug* in daily life from both genders and across ages.

^22^Derived from the initialism for “As Soon As Possible.”

^23^These transcriptions were confirmed by two phoneticists who do not speak AAE, who were not told what language the audio was, and who heard the utterances in isolation and were therefore not predisposed to a particular analysis.

^24^The reader may note that my preferred spelling, which helps to make the intensifier under discussion maximally distinct from the comparative phrase, is ranked number 10.

^25^While < v> or < h> might make more sense linguistically, < g> was much more common in the data.

^26^*Almost* perfectly, as variation in spelling (e.g., *hongry, hungryy* introduced some noise).

^27^I chose 10 or more tweets to ensure that there were sufficient observations to allow for a random term for username.

^28^Unsurprisingly, th-stopping, which is broadly productive in AAE, is not associated with loss of comparative morphology, and is not consistently significant across models.

^29^The most obvious and well-studied example is intensifier *-ass*, whose grammaticalization has been the subject of extensive study.

^30^It should be noted that for other varieties, it is indeed [æ], for instance, in New York City AAE.

^31^E.g.: https://soundcloud.com/blackmuzik/feelin-gooder-dennamug-feat.

^32^outchea < “*out here”*. Note, also, that the author is discussing what Labov refers to as the Sociolinguistic Monitor (Labov et al., 2011).

^33^dl < “*download.”*

^34^Heb. *kol-kli yutzar 'alaykha lo yitzlakh* lit. “all implements created over you will not succeed”.

^35^With TH-stopping.

^36^With orthographic representation of Southern AAE phonetic realization /ɔ/.

^37^Or, more bluntly, in media that would not allow orthographically inventive AAE through to publication without extensive editing.
